# Identification of Novel Protein Kinase Receptor Type 2 Inhibitors Using Pharmacophore and Structure-Based Virtual Screening

**DOI:** 10.3390/molecules23020453

**Published:** 2018-02-18

**Authors:** Josiane V. Cruz, Moysés F. A. Neto, Luciane B. Silva, Ryan da S. Ramos, Josivan da S. Costa, Davi S. B. Brasil, Cleison C. Lobato, Glauber V. da Costa, José Adolfo H. M. Bittencourt, Carlos H. T. P. da Silva, Franco H. A. Leite, Cleydson B. R. Santos

**Affiliations:** 1 Postgraduate Program in Pharmaceutical Sciences, Federal University of Amapá, Macapá, Amapá 68902-280, Brazil; 2 Laboratory of Modeling and Computational Chemistry, Federal University of Amapá, Macapá, Amapá 68902-280, Brazil; luciaanebarros@hotmail.com (L.B.S.); ryanquimico@hotmail.com (R.d.S.R.); josivan.chemistry@gmail.com (J.d.S.C.); cleyson.cl@gmail.com (C.C.L.); vilhenac@hotmail.com (G.V.d.C.); joseadolfo@unifap.br (J.A.H.M.B.); 3 Laboratory Molecular Modeling, Estadual University of Feira de Santana, Bahia 44036-900, Brazil; moysesfagundes@gmail.com (M.F.A.N.); fhpharm@gmail.com (F.H.A.L.); 4 Institute of Technology, Federal University of Pará, Pará, Amazon, Belém 66075-900, Brazil; dsbbrasil@ig.com.br; 5 Laboratory of Computational Pharmaceutical Chemistry, Faculty of Pharmaceutical Sciences of Ribeirão Preto, University of São Paulo, Ribeirão Preto, São Paulo 14040-020, Brazil; tomich@fcfrp.usp.br

**Keywords:** RIPK2, ponatinib, WEHI-345, virtual screening, rheumatoid arthritis, anti-inflammatory

## Abstract

The Protein Kinase Receptor type 2 (RIPK2) plays an important role in the pathogenesis of inflammatory diseases; it signals downstream of the NOD1 and NOD2 intracellular sensors and promotes a productive inflammatory response. However, excessive NOD2 signaling has been associated with various diseases, including sarcoidosis and inflammatory arthritis; the pharmacological inhibition of RIPK2 is an affinity strategy that demonstrates an increased expression of pro-inflammatory secretion activity. In this study, a pharmacophoric model based on the crystallographic pose of ponatinib, a potent RIPK2 inhibitor, and 30 other ones selected from the BindingDB repository database, was built. Compounds were selected based on the available ZINC compounds database and in silico predictions of their pharmacokinetic, toxicity and potential biological activity. Molecular docking was performed to identify the probable interactions of the compounds as well as their binding affinity with RIPK2. The compounds were analyzed to ponatinib and WEHI-345, which also used as a control. At least one of the compounds exhibited suitable pharmacokinetic properties, low toxicity and an interesting binding affinity and high fitness compared with the crystallographic pose of WEHI-345 in complex with RIPK2. This compound also possessed suitable synthetic accessibility, rendering it a potential and very promising RIPK2 inhibitor to be further investigated in regards to different diseases, particularly inflammatory ones.

## 1. Introduction

The protein kinase receptor type 2 (RIPK2) plays an essential role in the immune response and has been suggested to be a target in inflammatory diseases such as Crohn’s disease, inflammatory bowel disease, asthma and arthritis [1]. RIPK2 signals downstream of the NOD1 and NOD2 intracellular sensors promote a productive inflammatory response. However, excessive NOD2 signaling has been associated with various diseases including sarcoidosis and inflammatory arthritis; the pharmacological inhibition of RIPK2 is an affinity strategy that demonstrates an increased expression of pro-inflammatory secretion activity [1].

**Figure 1:**
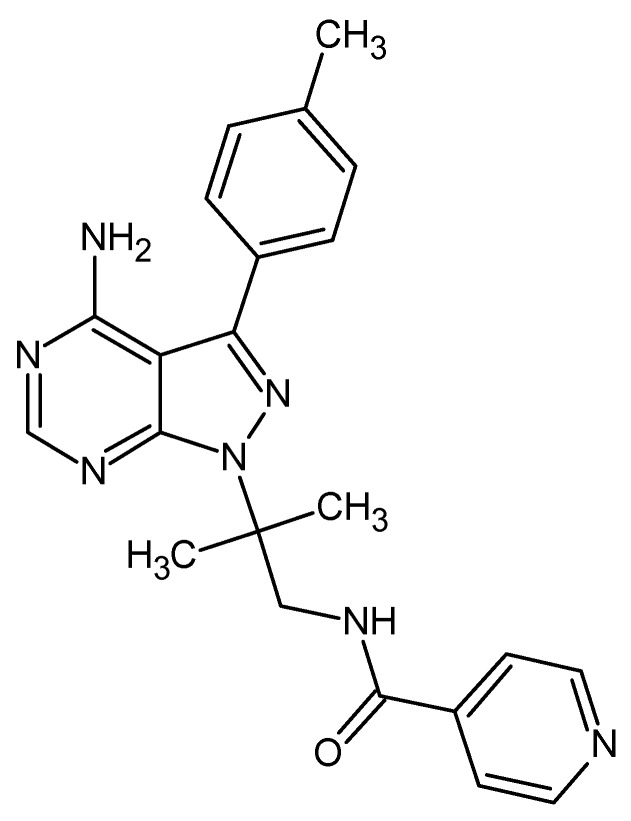
2D chemical structure of compound WEHI-345.

Drugs that inhibit RIPK2 may be quite effective at treating many different inflammatory diseases. According to Canning et al. [2], the compound ponatinib provided a structural base, when identifying an allosteric site, for the development of new inhibitors at this target. Characterization of ponatinib reveals desirable clinical features in treatment with tyrosine kinase inhibitors and a small molecule inhibitor of said protein [3]. However, because ponatinib is associated with side effects, has ruled against its consumption. Its dermatological side effects include rashes, erythematous and dermatitis, dry skin and erythema nodosum [4]. WEHI-345, shown in Figure 1, has been identified as a potent and selective inhibitor for RIPK2 [5]. WEHI-345 is an ATP analog and binds to the ATP binding pocket of RIPK2, IC_50_: 0.13 μM [5].

This study presents the design of novel potential drugs with anti-inflammatory activity in rheumatoid arthritis. Pharmacophore- and structure-based virtual screening approaches have been employed, as in similar studies conducted by Cichero et al. [6,7] and Liessi et al. [8]. In this analysis, the ponatinib as a template as well as 30 reported RIPK2 inhibitors, were selected from the Protein Data Bank (PDB, code 4C8B) and the BindingDB web server, respectively. We used the ZINC compounds database and predicted the in silico pharmacokinetic and toxicological properties of all of the compounds using virtual screening. This methodology was also employed by Leung and Ma [9] and Shoichet [10]. Furthermore, we assessed the potential biological activity for all of the novel compounds and the main enzyme-inhibitor interactions and binding affinity (kcal/mol). Yang et al. [11] and Kitchen et al. [12] also followed this technique. At least one of the compounds exhibited suitable pharmacokinetic properties, low toxicity and an interesting binding affinity and high fitness compared with the crystallographic pose (conformation + orientation) of WEHI-345 in complex with RIPK2. This compound also possesses suitable synthetic accessibility, rendering it a potential and very promising RIPK2 inhibitor to be further investigated with respect to different diseases, particularly inflammatory ones.

## 2. Results and Discussion

### 2.1. Pharmacophore Perception

**Figure 2:**
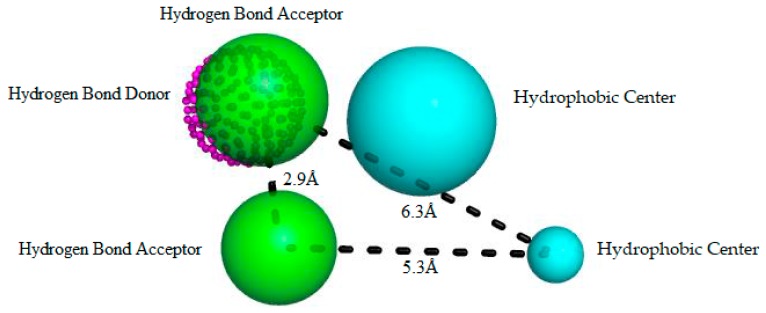
Best pharmacophore model here obtained for RIPK2 inhibitors. This model has two hydrophobic centers (cyan spheres), two hydrogen bond acceptors (green spheres) and a hydrogen bond donor (magenta dotted sphere). The size of the beads varies according to the tolerance radius calculated using GALAHAD. All the distances are measured in Angstroms.

We used the GALAHAD software [13] to generate pharmacophoric models based on known RIPK2 inhibitors (Figure 2). We allowed their torsional angles to vary, consistent with work by different authors [13,14,15]. We analyzed different sets and arrays of features for each pharmacophoric model that was generated (Table 1). Among the 10 pharmacophore models that we generated, five were discarded based on deformation energy criteria (<100 kcal mol).

Comparison of the Pareto indices revealed that they were statistically equivalent, although Model 07 indicated a high index of discrimination of the expected molecules (specificity > 5) compared with each model. Despite this fact, Model 07 did not satisfy the active training set as well as Model 03. Considering that the Mol_qry values reflect the agreement observed between the tuplet query and the hypermolecule generated, several studies have suggested that this parameter can be used to select the best pharmacophoric models. However, useful models can also have low Mol_qry values [16].

**Figure 3:**
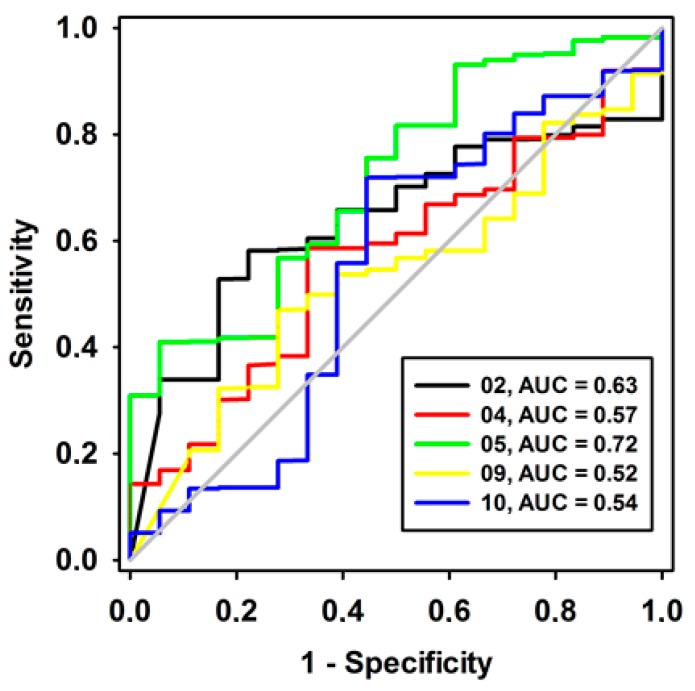
ROC curves of pharmacophore models here investigated, containing low strain energy. The diagonal line represents a model that would not be better than a randomic one (AUC < 0.5).

To circumvent this limitation, a well-established approach was used to select favorable pharmacophoric models. We relied on the ability to differentiate true binders from false positives [13]. Using such a strategy, we identified the pharmacophore model with the highest specificity and sensitivity and the ability to detect/select RIPK2 inhibitors instead of congenital molecules lacking activity. A dataset containing 17 RIPK2 inhibitors and 850 false positives was used to build ROC curves and to analyze the respective areas under the curve (AUC-ROC) using the QFIT value (0–100) (UNITY module alignment result, implemented in SYBYL-X 2.0 software) [17]. An AUC-ROC equal to 1.0 would be found in a model with impeccable specificity and sensitivity, and AUC = 0.5 would be associated with models with a poorer selection ability that was more pronounced than a random one. AUC > 0.70 may be considered to be a moderate predictive ability [18,19] (Figure 3). Therefore, Model 05 (AUC = 0.72) was selected as the most reliable pharmacophore for further analysis (Figure 2).

In Figure 2, Model 05 has two hydrophobic centers (cyan spheres), two hydrogen bond acceptors (green spheres) and one hydrogen bond donor (dotted magenta spheres). As observed across the kinase family, RIPK2 is characterized by a conserved domain; Glu66 and Asp164 residues allow hydrogen bonds with donor groups, and they are oriented towards a hydrophobic arrangement that offers an opportunity to optimize the selectivity of inhibitors [20].

Several authors have reported the importance of the polar interactions established by 25 residues for binding affinity; they have also noted that Ser176 plays an important role in modulating RIPK2 activity [2,5]. Among the common functional groups of molecules aligned generation of a common pharmacophoric pattern, the hydrophobic and hydrogen bond donor and/or acceptor ones can interact with the kinase domain, such as observed in Model 05.

**Figure 4:**
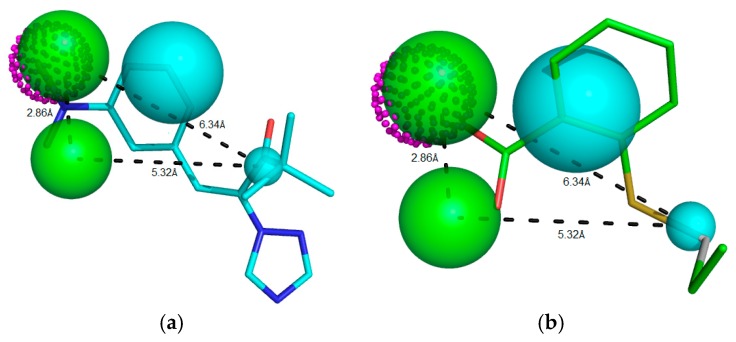
Potent RIPK2 inhibitor (**a**) IC_50_ = 0.90 μM, Mol_qry value = 51.35; and (**b**) weak RIPK2 inhibitor IC_50_ = 1.40 μM, Mol_qry value = 04.10 superimposed to the pharmacophoric Model 05, where green beads/spheres represents Hbond acceptor groups, magenta doted sphere represents Hbond donor groups, while hydrophobic ones are shown in cyan spheres. The size of the beads varies according to the tolerance radius calculated using GALAHAD. All the distances are measured in angstroms.

In addition to the predictive ability of the pharmacophoric model to recognize active compounds and false positives, model power measurement is essential information for virtual screening of new potential RIPK2 inhibitors. We assessed the ability of Model 05 to recognize inhibitors according to potency. By analyzing the conserved domain of kinases, we visualized the requirement of inhibitors containing hydrogen bond donor and acceptor groups and hydrophobic ones. Although compounds satisfied these pharmacophoric requirements to be potent RIPK2 inhibitors (Figure 4a), there was weak alignment between the inhibitor and Model 05 (Figure 4b). Despite the lack of a pharmacophore model for RIPK2 being reported thus far, the search for compounds fitting the pharmacophoric model that can make polar interactions with conserved catalytic residues increases the possibility of finding new hits. These compounds must possess characteristics that can be recognized by the active domain, and research is exploring the chemical diversity by enlarging the chemical space known thus far.

Once the Model 05 was chosen as the more reliable pharmacophoric one, virtual screening simulations we carried out virtual screening simulations in the ZINC compounds database [21] and found 1637 compounds with molecular groups that fit such a pharmacophore pattern. Thereafter, the pharmacokinetic predictions were subsequently performed for all of the compounds that we screened; 871 compounds were filtered/selected at this stage of the design process. In sequence, toxicological analyses were carried out using the next filter Derek 10.0.2 [22]; 414 “survivor” compounds were obtained.

### 2.2. Prediction of Activity Spectra for Substances

Prediction of potential biological activity was performed using the Prediction of Activity Spectral for Substances (PASS) [23] web server, which resulted in 29 selected compounds. Table 2 lists the characteristics/activity that we considered: anti-inflammatory activity, kinase inhibitor, autoimmune disease and treatment of rheumatoid arthritis. Values of Pa and Pi varying from 0 to 1 refer to the mean probability of being active or inactive, respectively. Estimates of the biological activity using PASS were related to aspects of similarity with other bioactive substances [24]. 

According to Table 2, the compounds **ZINC69349685**, **ZINC69349687**, **ZINC69431616**, **ZINC69431621** (with autoimmune and/or anti-inflammatory activities) and **ZINC91072217** (kinase inhibitor), all with Pa ˃ 0.5, were associated with the highest possibilities of being similar to other known bioactive compounds [25]. However, other compounds with Pa < 0.5 have also been selected to the next steps of the design process, when Pa ˃ Pi, such as considered by Rodrigues and Silva [26].

### 2.3. Molecular Docking: Molecular Interactions of the Selected Compounds

**Figure 5:**
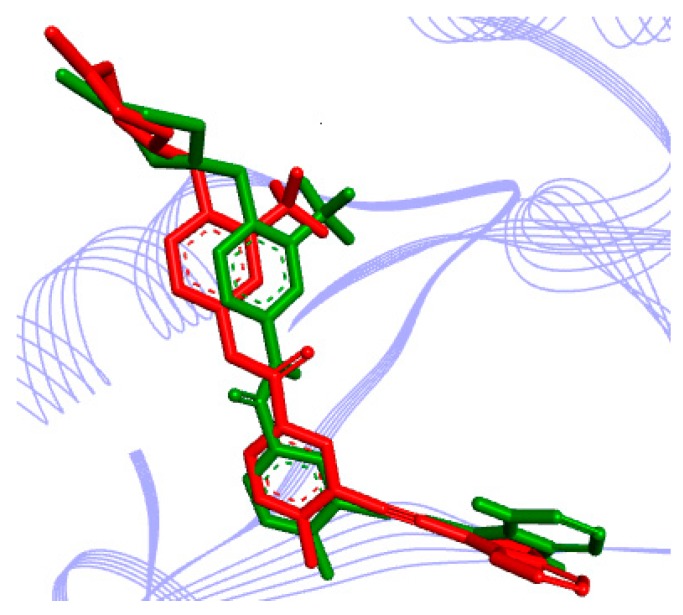
Result of validation for a known potent RIPK2 inhibitor, ponatinib, inside the enzyme active site (derived from PDB ID 4C8B), obtained with the AutoDock software: in red, the crystallographic pose; in green, the top-ranked docking pose. Inhibitor is represented in stick, whereas the RIPK2 active site is represented as a Ribbons diagram (in lines).

To validate the molecular docking approach that we used, the crystallographic pose of ponatinib, derived from the RPIK2-ponatinib complex structure (PDB ID 4C8B), and the top-ranked docking pose that we obtained were compared. The results are shown in Figure 5, which reveals the superposition of the two binding poses of ponatinib inside the RIPK2 binding site. This superposition results in a RMSD of superposition of 0.77. This result is below the well-established tolerance level of 2.0 Å, as has been reported by Hevener et al. and others [27,28].

In Table 3, only the interactions of RIPK2 with the crystallographic ponatinib or WEHI-345 are listed. Interactions of RIPK2 and the potential inhibitors screened here are listed in Table 4.

**Figure 6:**
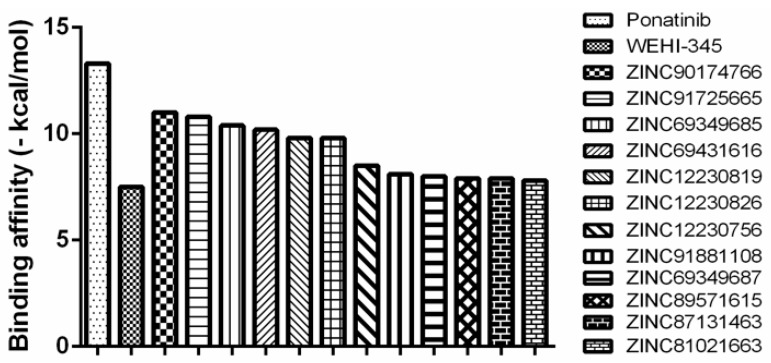
Binding affinity provided by the AutoDock/Vina software for the potent RIPK2 inihibitor and here used as a template—ponatinib, as well as for the control compound—WEHI-345 and the compounds obtained using a virtual screening approach, regarding RIPK2.

In Table 4, we list the binding affinity values calculated using AutoDock for the best-ranked compounds selected with the virtual screening approach [29]. The binding affinity values ranged from −7.80 kcal/mol (for **ZINC81021663**) to −11.00 kcal/mol (for **ZINC90174766**) relative to RIPK2. In Figure 6, we show all the compounds with interesting potential affinity for RIPK2 and values similar to the observed for ponatinib and WEHI-345.

**Figure 7:**
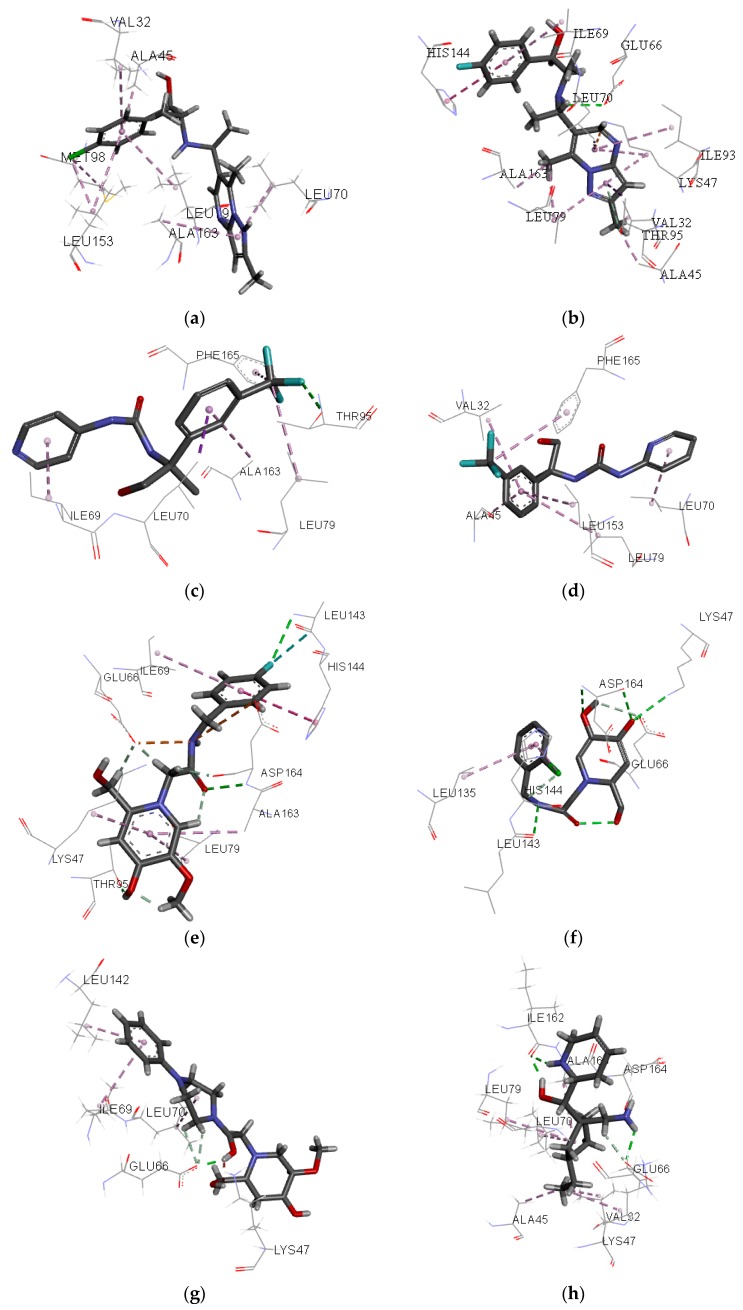

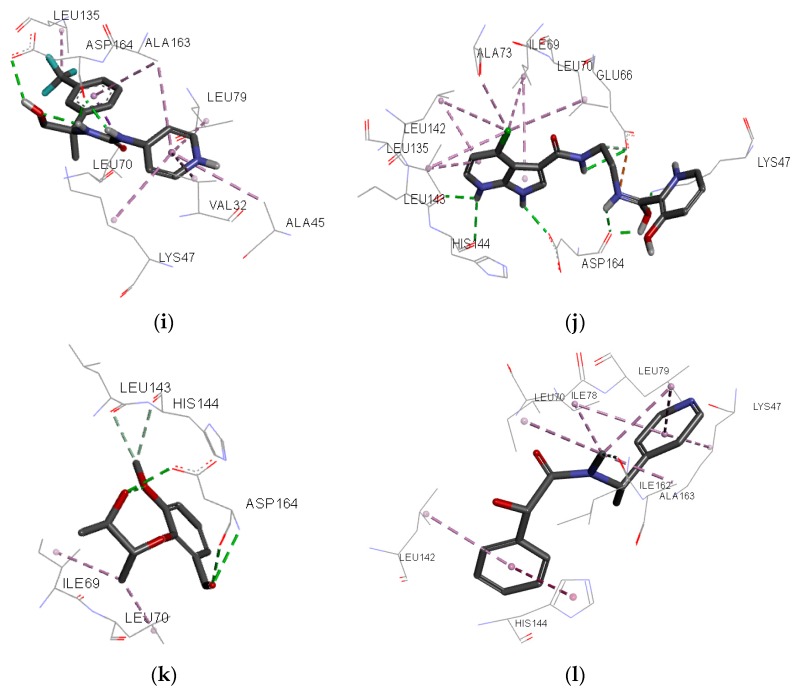
Individual net of interactions and contacts between RIPK2 (from PDB ID 4C8B) and the following compounds: (**a**) **ZINC90174766**; (**b**) **ZINC91725665**; (**c**) **ZINC69349685**; (**d**) **ZINC69431616**; (**e**) **ZINC12230819**; (**f**) **ZINC12230826**; (**g**) **ZINC12230756**; (**h**) **ZINC91781108**; (**i**) **ZINC69349687**; (**j**) **ZINC89571615**; (**k**) **ZINC87131463**; (**l**) **ZINC81021663**, thus calculated using the AutoDock/Vina software.

**Figure 8:**
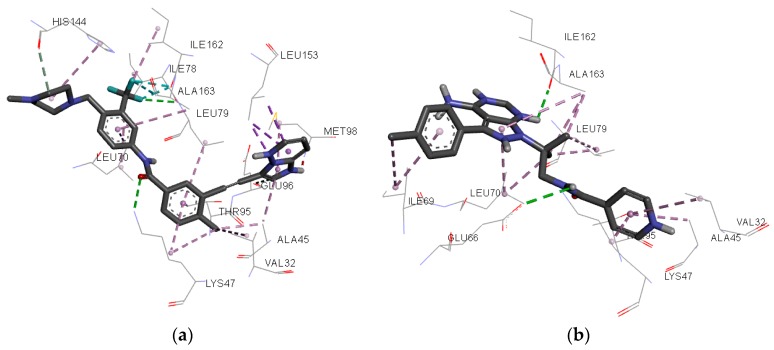
Individual net of interactions and contacts between RIPK2 (from PDB ID 4C8B) and (**a**) Ponatinib and (**b**) WEHI-345, thus calculated using the AutoDock/Vina software.

**Figure 9:**
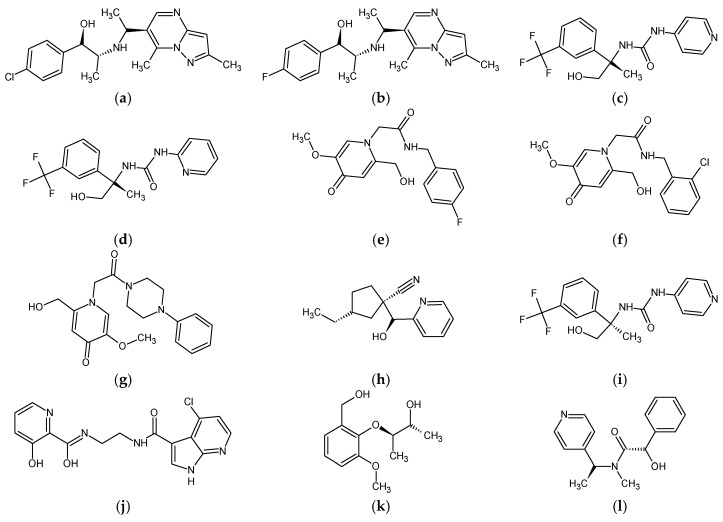
2D chemical structures of 12 predicted molecules selected with molecular coupling: (**a**) **ZINC90174766**; (**b**) **ZINC91725665**; (**c**) **ZINC69349685**; (**d**) **ZINC69431616**; (**e**) **ZINC12230819**; (**f**) **ZINC12230826**; (**g**) **ZINC12230756**; (**h**) **ZINC91881108**; (**i**) **ZINC69349687**; (**j**) **ZINC89571615**; (**k**) **ZINC87131463** and (**l**) **ZINC81021663**.

A complete net of interactions and contacts between RIPK2 and the template compound, ponatinib, is shown in Figure 7; a similar net of molecular interactions between RIPK2 and the compounds selected after virtual screening, followed by docking single, is shown in Figure 8. Most of the interactions predicted with docking for the 12 compounds (Figure 9) were the same as those observed for ponatinib (Table 4).

**ZINC90174766** interacts by π-alkyl with the ALA163, LEU79 and ALA45 residues from RIPK2, and **ZINC91725665** has two π-alkyl interactions with LYS47 and two alkyl interactions, with VAL32 and ALA45 from the same human enzyme structure. **ZINC69349685** interacts only via the π-alkyl bond with ALA163 from RIPK2, and **ZINC69431616** has two π-alkyl interactions, with ALA45 and LEU79 from the same enzyme structure. For **ZINC12230819**, two hydrogen bond interactions with LYS47 from RIPK2 were observed, and **ZINC12230826** interacts with RIPK2 LYS47 via two hydrogen bonds and one π-alkyl with HIS144. **ZINC12230756** has two hydrogen bonds with LYS47 from RIPK2 and three π-alkyl interactions (with ALA45, LYS47 and LEU79) and one alkyl interaction with LEU70 from the same human enzyme structure. **ZINC91881108** has four alkyl interactions with RIPK2, via ALA45, LEU70, VAL32 and LYS47. **ZINC69349687** interacts via π-alkyl bonds with ALA163, ALA45, LYS47, LEU79 and ALA163 from RIPK2. **ZINC89571615** has two hydrogen bonds (LYS47 and HIS144) and an alkyl interaction (LEU70 from the same enzyme structure. **ZINC87131463** interacts via hydrogen bond with RIPK2 HIS144 and an alkyl interaction with LEU70; **ZINC81021663** has Alkyl bonds with LYS47 and LEU70. 

Regarding the compound WEHI-345, all of the interactions observed with RIPK2 were also present in the RIPK2-inihibitor complex formed with the compounds selected. **ZINC90174766** has four π-alkyl interactions with LEU70, ALA163, ALA45 and VAL32 from human RIPK2, and **ZIN91725665** has a hydrogen bond with GLU66 and four π-alkyl interactions (with LYS47, ILE69, LEU70 and LYS47) and two alkyl interactions with LEU79 and ALA163 from the same enzyme structure. **ZINC69349685** has an alkyl interaction with LEU79 and two π-alkyl interactions with ALA163 and ILE69 from RIPK2. Docking for **ZINC69431616** reveals three π-alkyl interactions with VAL32, ALA45 and LEU70 from the same enzyme structure. For **ZINC12230819**, a hydrogen bond is observed with RIPK2 GLU66, as well as a π-alkyl with ILE69. **ZINC12230826** has two hydrogen bonds with GLU66, and **ZINC12230756** interacts via hydrogen bonds with GLU66 from RIPK2 and one Alkyl interaction with LEU70 as well as four π-alkyl bonds with ILE69, VAL32, ALA45 and LYS47 from the same enzyme structure. **ZINC91881108** has hydrogen bonds with ILE162 and GLU66 from RIPK2 and three other alkyl interactions with ALA163, LEU70 and LEU79. **ZINC69349687** interacts via five π-alkyl bonds with ALA163, VAL32, ALA45, LYS47 and ALA163. For **ZINC89571615**, two hydrogen bonds are observed with RIPK2 GLU66, one Pi-alkyl interaction with ILE69, and one alkyl interaction with the same amino acid residue. **ZINC87131463** has two alkyl interactions with RIPK2, via ILE69 and LEU70, and **ZINC81021663** has alkyl interactions with LEU70, LEU79 and ALA163 and one hydrogen bond with ILE162 from the same enzyme structure.

### 2.4. Molecular Overlap of Screened Compounds with Ponatinib and WEHI-345

Similarity analysis of the molecular overlap of the compounds selected, after molecular docking, with ponatinib WEHI-345 can be observed for the most promising compounds based on the data in Table 5 and Table 6, respectively. 100% steric (100ste) and 100% electronic (100elt), 60% steric (60est) and 40% electronic (40elt) and 50% steric and electronic (50est/elt) similarities to ponatinib are listed in Table 5, and the same pairs of similarities compared with WEHI-345 are listed in Table 6.

Our analysis regarding the template compound (ponatinib) revealed the compounds **ZINC90174766**, **ZINC91725665** (100ste), **ZINC91881108** (100elt) as exhibiting the highest values of similarity of the molecular overlap (Table 5). According to Costa et al. [30], the closer the value of is to 1 the greater the degree of structural similarity between the compounds.

Analysis regarding to the WEHI-345 revealed that the compounds **ZINC12230819**, **ZINC91881108** (100elt) and **ZINC81021663** (60est/40elt; 40est/60elt; 50est/elt) exhibited the largest values of similarity of overlay compared with the other ones (Table 6).

It is worth noting that the compound **ZINC91881108** stood out with the highest value for similarity of molecular overlap, based on a 100% electronic analysis, compared with ponatinib and WEHI-345.

### 2.5. Overlap of Potential RIPK2 Inhibitors Regarding the Pharmacophoric Model

We evaluated the concordance between the pharmacophoric features and the groups found in these new molecules. Then, by superimposing them on the model, in accordance with the respective Cartesian coordinates and radii, we observed that only 10 of the 15 compounds successfully overlapped with the model. The overlapping score (QFIT) ranged from 12.21–52.28, as indicated in Table 7.

**Figure 10:**
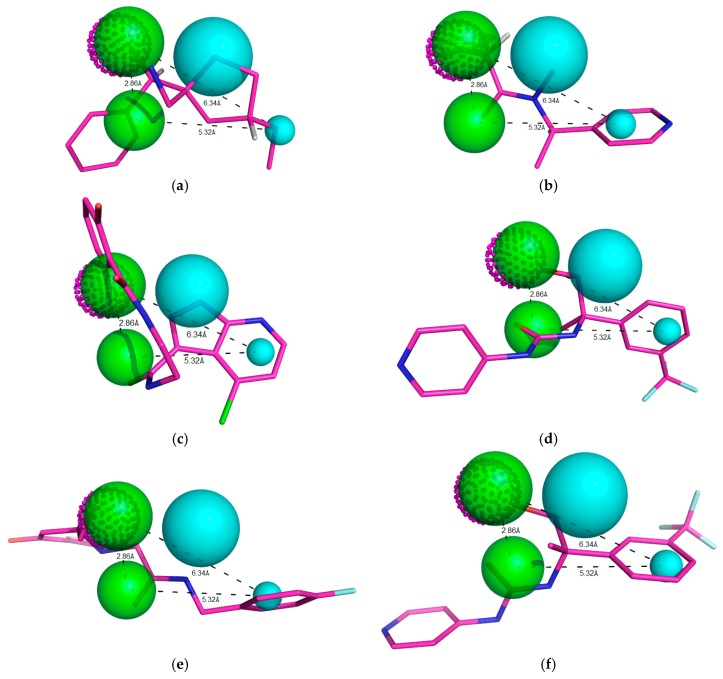

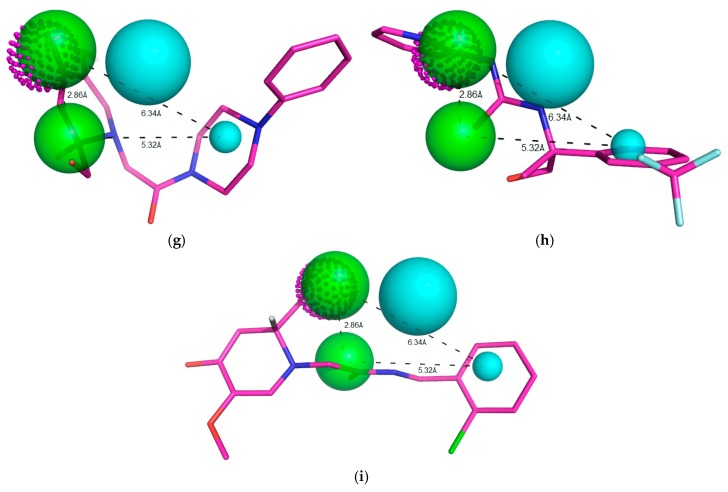
Representation of the ten compounds that fit to the pharmacophoric model, with QFIT > 0. Green beads/spheres represent H-bond acceptor groups, magenta dotted spheres represent H-bond donor groups, while hydrophobic centers are shown in cyan spheres. Size of the beads varies according to the tolerance radius calculated using GALAHAD. All the distances are measured in angstroms (**a**) **ZINC91881108**; (**b**) **ZINC81021663**; (**c**) **ZINC89571615**; (**d**) **ZINC69349687**; (**e**) **ZINC12230819**; (**f**) **ZINC69349685**; (**g**) **ZINC12230756**; (**h**) **ZINC69431616** and (**i**) **ZINC12230826**.

**ZINC91881108** exhibited the best value of QFIT (52.28%). The aim of finding potential molecules that overlap with the best model is to identify a promising compound that possesses the essential stereo-electronic requirements for RIPK2 inhibition that is described/explained by the pharmacophoric model. Figure 10 shows the 20 compounds aligned according to the established Cartesian coordinates.

### 2.6. Analysis of the Physicochemical and Toxicological Properties of the Compounds

Pharmacokinetic properties are strictly related to the administration of a drug, and they involve aspects of absorption, distribution, metabolism and excretion in the organism [31].

In Table 8, data are listed for eight molecular descriptors for analysis of 10 selected compounds. These data include the following parameters: drug similarity (number of stars/violations), Lipinski rule of five, percentage of human absorption (HOA%), Caco-2 and MDCK cell permeation, hydrophilic/lipophilic balance (Qplog Po/w), central nervous system (CNS) activity and blood-brain barrier permeability (Qplog BBB).

The parameter “stars” indicates descriptors that are outside (violations) the optimum range of values described for 95% of known drugs contained in the QikProp database. In such analyses, all of the selected compounds exhibited values equal to zero (no violations), indicating important similarity with commercially available drugs. On the other hand, ponatinib exhibited violations.

The Lipinski rule of five represents a well-established form of simple limits for absorption and permeability of drugs. In Table 8, data show that all the compounds selected exhibited interesting increases in oral absorption in the organism. The percentage of human oral absorption (HOA%) was considered to be high; all of the compounds exhibited values higher than 80%. On the other hand, ponatinib exhibited a value of 73%.

The apparent perception of absorption of drugs in the gastrointestinal tract using Caco-2 and MDCK cells (ACP and AMP, respectively) was investigated for values <25 (low) and >500 (optimum). Most of the molecules listed in Table 8 exhibit excellent values, except for the compounds **ZINC12230819**, **ZINC12230826**, **ZINC89571615** and the template and control compounds (ponatinib and WEHI-345), which were considered to be intermediates.

The parameter established to indicate inactivity for penetration into the blood-brain barrier and consequent CNS activity includes values below 1 (CBrain/CBlood < 1). In this work, all the compounds exhibited values lower than 1. Ideally, these compounds can be thought as being inactive in the CNS and therefore immune to side effects in humans [32]. In considering the permeability of drugs into the CNS (calculating Qplog BBB)—negative values indicate a higher concentration of the compound in the blood than in the brain—our results indicate that the compounds we investigated only exhibited negative values. The parameter established as QPlog Po/w, with an optimum interval ranging from −2.0 to 6.5, is related to the bioavailability and permeability of the compounds through the membranes in the hydrophilic and lipophilic balance. To this end, all of the compounds that we investigated fell within the given limits (Table 8).

In the toxicological in silico investigation of the nine selected compounds, which was performed using DEREK Nexus software, potential toxicity (carcinogenicity, chromosomal damage, genotoxicity, hepatotoxicity, HERG channel inhibition, irritation, mutagenicity, reproduction toxicity, respiratory sensitization, skin sensitization, thyroid toxicity) was analyzed. We found that none of the compounds analyzed had a potential toxic. On the other hand, the template compound (ponatinib) exhibited a toxicophoric group (aryl piperazine) based on DEREK 10.0.2 Nexus software [22] analysis.

### 2.7. Prediction of Synthetic Accessibility

The synthetic accessibility of the compound **ZINC91881108** had a computed score of 5.01 (moderately difficult) (Table 9). This finding is similar to that of ponatinib as well as WEHI-345, leaving us to propose future synthesis and subsequent activity assays for such a compound.

### 2.8. Structure—Activity Relationship of the Promising Molecule

**Figure 11:**
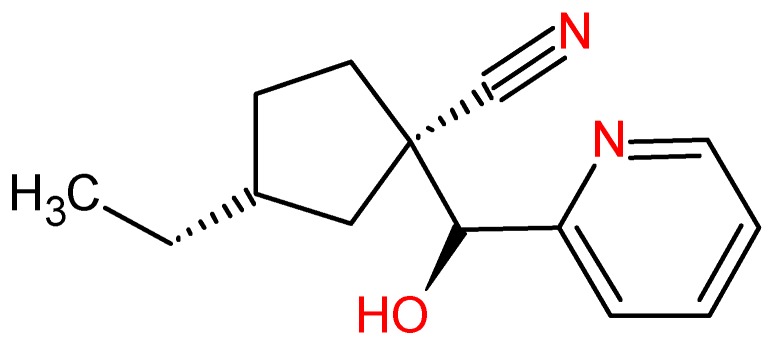
Promising molecule **ZINC91881108** obtained after virtual screening.

The biological response of a structurally specific drug depends necessarily on the identification of the active site and its spatial mutuality. Therefore, analysis of the pharmacophore cluster enables the stereo-electronic recognition that is fundamental to its pharmacological activity [33]. According to a study by Canning et al. [2] of the crystallographic pose of the amino acid residue interactions of the RIPK2 complexed to ponatinib, there are hydrogen bonds (interactions with Glu66A, Asp164A, Met98A) and hydrophobic interactions. Therefore, the pharmacophore (Figure 2) shares hydrophobic regions and the promising molecule **ZINC91881108** interacts with hydrogen bonds (Asp164 and Glu66) (Figure 11).

**Figure 12:**
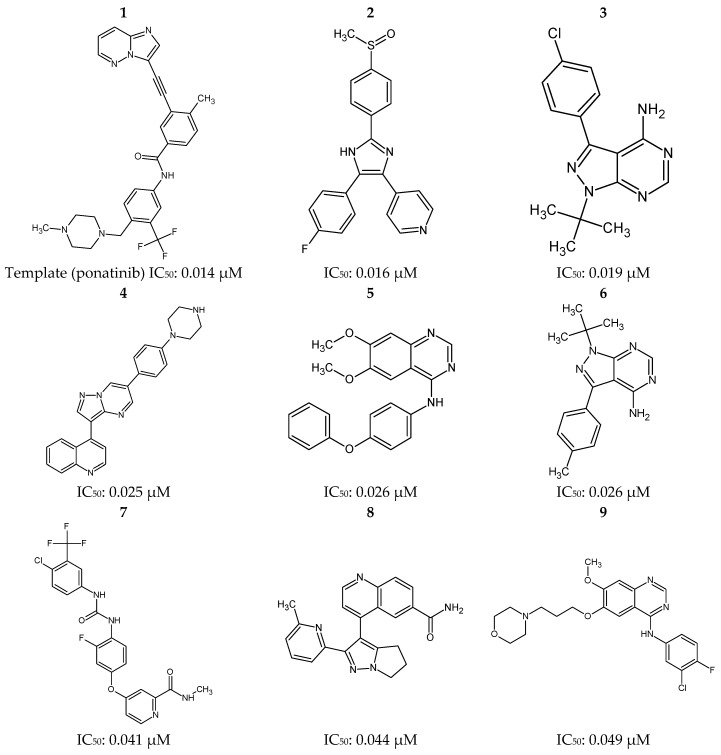

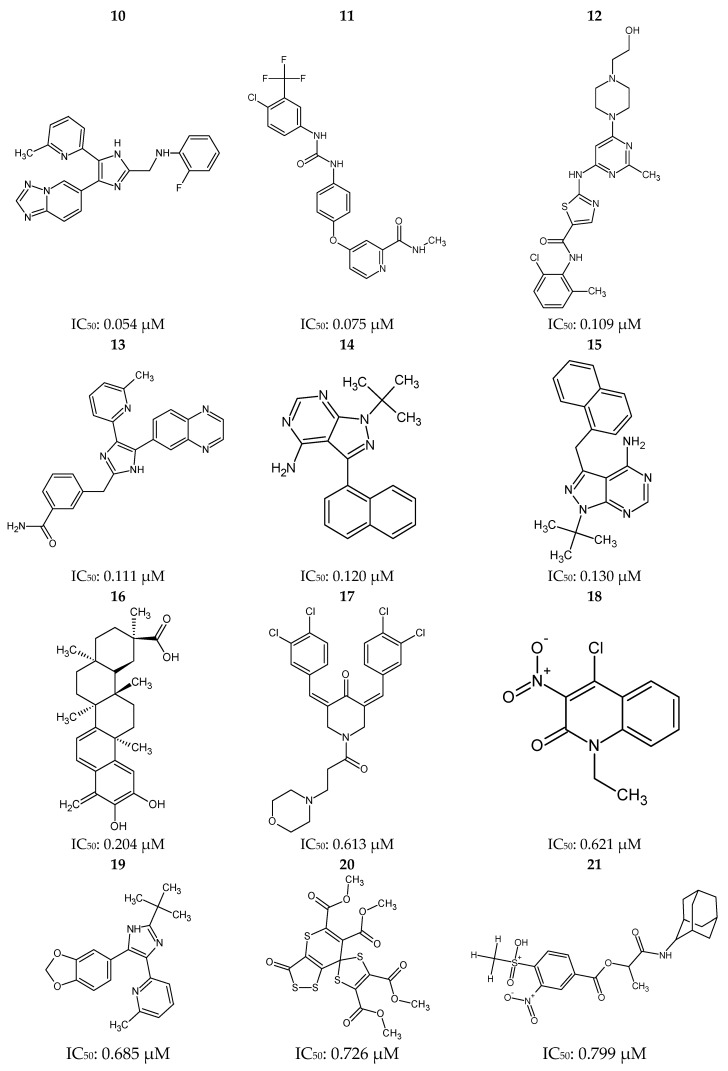

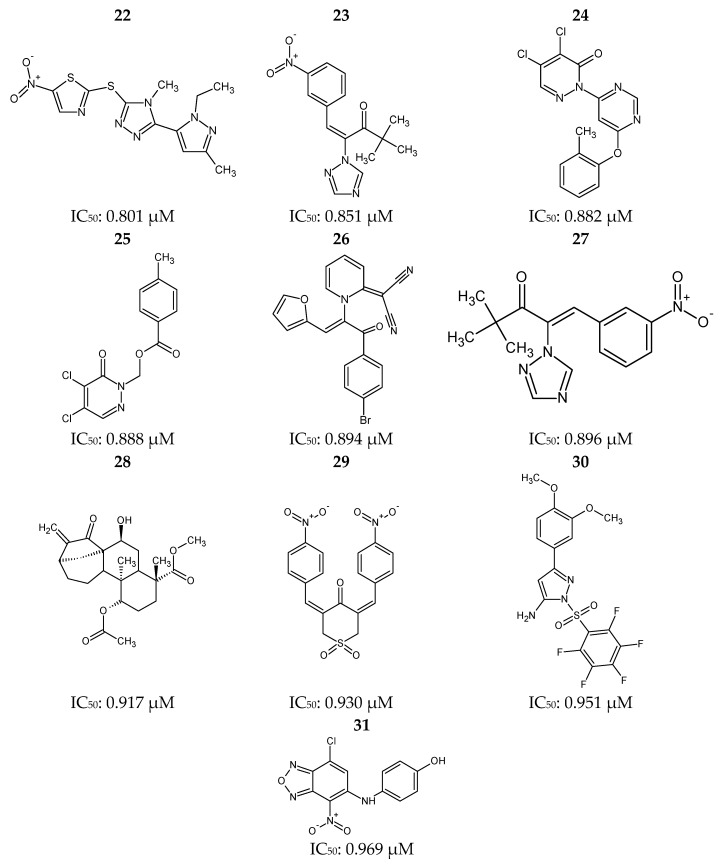
Ponatinib (**1**) and 30 most potent RIPK2 inhibitors (**2**–**31**) obtained from the Protein Data Bank (PDB) and the BindingDB database.

When analyzing ponatinib (Figure 12) (**1**) (compound template), we observed that it had regions characterized by an imidazole ring, which forms hydrogen bonds, and hydrophobically interacting pyridazine. In Figure 11, we show the molecule **ZINC91881108** possessing the pyridine derivative group, which has anti-inflammatory activities. This finding has been reported by Sondhi et al. [34] and Sangshetti et al. [35].

The primary amine present in the compound and the hydroxyl is an important group because it is capable of improving the solubility of the compound and modifying the chemical reactivity of the drug-receptor interaction [36].

## 3. Materials and Methods

### 3.1. Selection of Compounds

Selection of the compound ponatinib [2] and other inhibitors reported in literature was done from the Protein Data Bank (PDB) and the BindingDB (http://www.bindingdb.org) [37] web server, respectively (2D structures are shown in Figure 12), according to the lower IC_50_ values there reported (between 0.016 and 0.969 μM), which were considered limiting for this selection, such as observed in studies developed by Pereira and Costa et al. [30,38]. Such inhibitors are related to the Protein Kinase Receptor type 2—RIPK2, for which the structure of the potent inhibitor ponatinib is deposited in the PDB [39] in a complex with human RIPK2 (PDB ID 4C8B, at 2.75 Å resolution).

After definition of the small database to be built (ponatinib + 30 reported and potent RIPK2 inhibitors), the most reliable tautomers (at pH = 7.5) were selected for all the compounds, using the Marvin^®^ Sketch 16.9.5 software (https://www.chemaxon.com/) [2,40]. Subsequently, structures were converted to 3D format using CONCORD, with default parameters, thus implemmented on the SYBYL^®^-X 2.0 package [17]. All the structures were energy-minimized using Conjugate Gradient (CG) and a convergence criterion of 0.001 kcal/mol, using the Tripos [41] force field (with dielectric constant ε = 80.4 and maximum number of iterations = 50,000). Partial atomich charges were calculated using the Gasteiger-Hückel method [42], such as available on the SYBYL^®^-X 2.0 platform.

**Figure 13:**
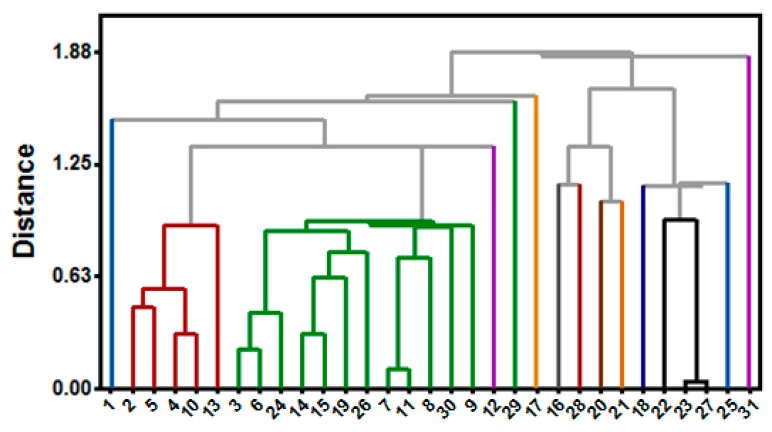
Dendrogram used to investigate chemical similarity of RIPK2 inhibitors.

In order to select the best set (the training set) of inhibitors able to generate a reliable pharmacophoric model, a chemical similarity study was carried out. Inhibitors were thus selected according to the chemical diversity, estimated using the web server ChemGPS-NP [43], so that the most potent compounds of each cluster were selected by hierarchical cluster analysis [44]. The first three principal components (PC1, PC2 and PC3) were used to construct the dendogram related to the chemical similarity study (Figure 13).

A dendogram was thus generated considering the centroid method and the Euclidean distance here used as a measurement parameters, and 85% (threshold) of similarity between the compounds [44]. In sequence, the representative one of each cluster that showed the highest biological activity was chosen to compose the so called “training set”, which was constructed from a dataset of 14 RIPK2 inhibitors (group A). IC_50_ values are: 0.014 μM (**1**), 0.016 μM (**2**), 0.019 μM (**3**), 0.109 μM (**12**), 0.204 μM (**16**), 0.613 μM (**17**), 0.621 μM (**18**), 0.726 μM (**20**), 0.799 μM (**21**), 0.801 μM (**22**), 0.888 μM (**25**), 0.917 μM (**28**), 0.930 μM (**29**) and 0.969 μM (**31**). 

A second dataset with 17 RIPK2 inhibitors (group B) was choosen, with IC_50_ values of: 0.025 μM (**4**), 0.026 μM (**5**), 0.026 μM (**6**), 0.041 μM (**7**), 0.044 μM (**8**), 0.049 μM (**9**), 0.054 μM (**10**), 0.075 μM (**11**), 0.111 μM (**13**), 0.120 μM (**14**), 0.130 μM (**15**), 0.685 μM (**19**), 0.851 μM (**23**), 0.882 μM (**24**), 0.894 μM (**26**), 0.896 μM (**27**) and 0.951 μM (**30**). Such compounds were here submitted to the same protocol of energy minimization and they were used only for the evaluation of the pharmacophoric models, here so called the “test set”.

### 3.2. Pharmacophore Modelling

The method here used to derive the pharmacophoric pattern is Genetic Algorithm with Linear Algorithm for Hypermelecular Alignment of Data Sets (GALAHAD), a module implemented in the SYBYL platform [45]. Inhibitors of the training set were flexibly superimposed in order to create hypermolecular alignments that mapped common pharmacophore characteristics. The genetic algorithm employed in this step starts with 80 conformations (population size) of each RIPK2 inhibitor that evolves through a maximum of 50 generations through standard genetic operators (mutation rate—Angle: 0.6, Conf: 0.3; mutation drop-Angle: 1.0, Conf: 1.0 and crossover rate-Angle: 1.0, Conf: 1.0), such as implemmented in the GALAHAD module from the SYBYL-X^®^ 2.0 package. Statistical parameters (deformation energy, PARETO, hydrogen bonds and steric) and pharmacophore (Mol_qry values) were used to evaluate the models. Models with deformation energy containing two orders of magnitude higher than the others were discarded.

#### 3.2.1. Evaluation of the Pharmacophoric Models

Pharmacophore models were first tested for their ability to differentiate true inhibitors from false positives, and they were constructed using the DUD-E server [46]. After, the test set database (17 false-positive RIPK2 inhibitors, with 850 compounds) was aligned to each pharmacophore model, using GALAHAD default parameters, and they were classified according to their Mol_qry values.

Operational Characteristic Receiver Curves (ROC) were then used to evaluate the specificity and sensitivity of each model. Next, the pharmacophoric models with AUC > 0.7 were probed by their ability to classify the inhibitors according to their potency. In this step, 34 RIPK2 inhibitors, the test set (17 inhibitors-Group B, see Figure 1) and 17 other inhibitors randomly selected (T1-T17), with IC_50_ values of 0.0063 μM (T1), 0.0079 μM (T2), 0.01 μM (T3), 0.012 μM (T4), 1.03 μM (T5), 1.1 μM (T6), 1.12 μM (T7), 1.15 μM (T8), 1.18 μM (T9), 1.19 μM (T10), 1.20 μM (T11), 1.23 μM (T12), 1.32 μM (T13), 1.35 μM (T14), 1.36 μM (T15), 1.36 μM (T16) and 1.4 μM (T17), which were not used in the model generation, were individually aligned to a model, such as implemented in the GALAHAD module. The Mol_qry values were then plotted versus the biological activity classes of the compounds (using the pIC50 = −logIC50 equation) and classified as the following: weak = 5.0 to 5.9; moderate = 6.0 to 6.9 and strong = 7.0 to 9.0. ROC curves of pharmacophore models were build via the SigmaPlot^®^ software [47].

#### 3.2.2. Selection of Novel and Potential RIPK2 Inhibitors from the ZINC Compounds Database

The ZINC compounds database is the largest one commercially available for virtual screening purposes, and it contains more than 14 million compounds [21]. After building of the most reliable pharmacophore model, thus validated using the GALAHAD approach, the ZINCPharmer (http://zincpharmer.csb.pitt.edu) [48] web server was used to search for compounds in the ZINC database, using the pharmacophoric model obtained and here used as a “probe“. For this step, we used some search filters, so that the maximum value of RMSD (Root Mean Square Deviation) employed was 0.3. In addition, interval values of 200 ≤ Molecular Weight ≤ 500 and 1 ≤ Rotational Connections ≤ 10, were here defined for predictions, as well as observed in studies of Birck et al. (2016) [49]. Therefore, in order to procedd with search for active compounds in the database, we considered the 3D coordinates described in Table 10, according to the respective pharmacophoric model generated.

### 3.3. Pharmacokinetic and Toxicological Predictions—ADME/Tox

Pharmacokinetic in silico analyses were performed for the selected molecules, using the QikProp software [50], where criteria such HOA (human oral absorption), PHOA (percentage of human oral absorption) and “stars”, which indicates the number of violations of properties values intervals reported for 95% known drugs, where considered such as described by Onguéné et al. (2014) [51].

Toxicity profile of the compounds was evaluated using the Deductive Estimation of Risk from Existing Knowledge (DEREK) 10.0.2 software [22]. We have considered DEREK alerts of toxicity involving the human species and also classified as plausible in mammals, but compounds containing any toxicophoric groups were also discarded. The DEREK software [22] makes the prediction of toxicity of the compounds in a qualitative way, is a specialist system that focuses attention on the toxic action of chemical compounds. The system performs this analysis based on implemented rules and depicts the relationship between a structural feature and a toxicophore group present in the compounds as possible inducers of certain types of toxicity. It is considered that in addition to toxicity DEREK can identify aspects related to carcinogenicity, mutagenicity, skin sensitization, irritation, teratogenicity and neurotoxicity [52].

### 3.4. Prediction of Activity Spectra for Substances (PASS)

Prediction of potential biological activity was performed through the PASS (Prediction of Activity Spectra for Substances) web server, at http://pharmaexpert.ru/passonline/predict) [24]. Inflammatory diseases and mechanisms associated were here considered, such as treatment of rheumatoid arthritis, autoimmune activity, kinase inhibitors, etc., according to studies of Volpini; Pedersoli-Mantoani et al. [25,53] Rodrigues and Silva [26].

### 3.5. Docking Procedures

For molecular docking here performed, the crystallographic structure of the RIPK2 derived form RIPK2-ponatinib complex structure (PDB ID 4C8B, at 2.75Å resolution) [2] was used. The enzyme structure was prepared by removing water and ligands, subsequently adding hydrogen atoms.

Compounds here selected using a virtual screening approach were submitted to the docking single simulations, using the PyRx 0.8 software, with further energy minimization [54]. Ponatinib [2] and WEHI-345, respectively a potent and a selective RIPK2 inhibitor [5], were used as template and control inhibitors. Docking calculations were performed using the AutoDock 4.2/Vina 1.1.2 [55] software, with default parameters of the genetic algorithm (with population size of 150), maximum number of evaluations of 250,000, maximum number of generations of 27,000 and crossing rate of 0.8. Interactions between the inhibitors and RIPK2 were visualized using the Discovery Studio 4.1 [56] software, with default parameters. We have used a grid box of *x* = 56, *y* = 28 and *z* = 24 coordinates, centered at *x* = 14.254, *y* = 2.632 and *z* = 23.776. Ten docking runs were considered and the ten poses were analyzed.

### 3.6. Molecular Overlay—Molecular Overlay

Molecular Overlay is used to overlap two or more molecules using a variety of features that includes, in addition to other aspects, alignment by a combination of steric (ste) and electrostatic (elt) fields [56]. For this purpose, analyses of the steric and electronic overlaps were predicted using the Discovery Studio 4.1 software [56], considering 100% ste, 100% elt, 60% ste/40% elt, 40% ste/60% elt and 50% ste/elt, according to studies of Costa et al. (2017) [30] between the RIPK2 inhibitors and Ponatinib. In sequence, similar protocol was employed using WEHI-345.

### 3.7. Alignment Overlap of Inhibitors with the Pharmacophoric Model

We have used the methodology implemented in the CHEMGPS-NP (http://chemgps.bmc.uu.se) web server to evaluate the quality of the alignment of each inhibitor. The QFIT value associated to the overlap means the degree of alignment ranging from 0 to 100, and it is calculated automatically to select the most promising models [57].

### 3.8. Sylvia—Estimation of the Synthetic Accessibility of Organic

In this step, the Sylvia 1.4 [58] server was used to estimate the synthetic viability of the compounds here investigated. For such prediction, the promising compound was compared with the template one (ponatinib) as well as to the control (WEHI-345). For analysis, it is considered that the estimation of synthetic accessibility provides a number between 1—for easily synthesized compounds, and 10—for compounds that are difficult to synthesize, according to studies developed by Ferreira et al. [59].

## 4. Conclusions

We indicate compound **ZINC91881108**, discovered using a virtual screening approach from the ZINC compounds database as a promising RIPK2 inhibitor, with further interest in control of inflammatory diseases. Pa » Pi is observed for such compound, besides a potential anti-inflammatory activity. Analysis of molecular docking for this compound reveals a potential higher binding affinity, in comparison to WEHI-345. In a 100% electronic analysis when overlapping of **ZINC91881108** with ponatinib or WEHI-345, such compound stand out for having a highest value for similarity of overlap. Thus, this compound has the best score of stereoelectronic overlap, when being sorted. The importance of this present work is evident because, regarding to structure-activity relationships (SAR), the steric arrangement is of fundamental relevance for the drug-enzyme interaction. In addition, the electronic aspects are strictly related to the electronic density and physicochemical properties and polar interactions associated. Compound **ZINC91881108** shows suitable pharmacokinetic properties, when compared to the template compounds—RIPK2. Also, such compound does not contain any toxicophoric groups, such as analyzed using the DEREK software. Regarding synthetic accessibility, the said compound **ZINC91881108** is predicted in silico to be moderately difficult to prepare.

## Figures and Tables

**Table 1:** Features of each pharmacophoric model generated using GALAHAD.

Model	Specificity	N_Hits	Features	Pareto	Energy (kcal/mol)	Sterics	HBond	Mol_QRY
01	2.399	03	07	00	1215.88	541.80	57.00	3.29
02	3.069	01	09	00	90.97	532.10	54.00	2.22
03	3.875	00	06	00	303, 52.40	537.80	55.30	5.78
04	3.676	01	05	00	31.35	529.70	55.30	0.58
05	2.993	03	05	00	30.33	472.30	54.60	2.25
06	1.900	03	04	00	148.83	533.80	52.90	2.67
07	5.526	00	08	00	187, 147.79	547.20	56.80	0.53
08	3.939	01	06	00	4453, 411.50	549.30	56.50	1.67
09	3.068	03	05	00	45.49	536.90	54.60	0.22
10	3.082	04	04	00	72.56	514.90	55.20	0.80

**Table 2:** Prediction of biological activity of substances (PASS).

Compound (ZINC Code)	Biological Activity	Pa	Pi
**ZINC69349685**	Autoimmune	0.84	0.005
	Anti-inflammatory	0.538	0.046
	Treatment of rheumatoid arthritis	0.434	0.019
	Kinase Inhibitor	0.316	0.128
**ZINC69349687**	Autoimmune	0.784	0.005
	Anti-inflamatória	0.538	0.046
	Treatment of rheumatoid arthritis	0.434	0.019
	Kinase Inhibitor	0.316	0.128
**ZINC69431616**	Autoimmune	0.790	0.005
	Anti-inflammatory	0.573	0.038
	Treatment of rheumatoid arthritis	0.436	0.019
	Kinase Inhibitor	0.133	0.104
**ZINC69431621**	Autoimmune	0.790	0.005
	Anti-inflammatory	0.573	0.038
	Treatment of rheumatoid arthritis	0.436	0.019
	Kinase Inhibitor	0.133	0.104
**ZINC91072217**	Kinase Inhibitor	0.572	0.023
	Anti-inflammatory	0.259	0.203

**Table 3:** Interactions between RIPK2 (PDB 4C8B) and ponatinib or WEHI-345, obtained using the AutoDock/Vina software are shown.

Compound	Amino Acid	Type	Distance (Å)	Binding Affinity (in kcal/mol)
Ponatinib	LYS47	Hydrogen Bond	3.09	−13.30
	HIS144	Hydrogen Bond	3.12	
	ALA45	Alkyl	3.33	
	VAL32	Alkyl	4.98	
	LYS47	Alkyl	4.80	
	LEU70	Alkyl	5.42	
	HIS144	π-Alkyl	4.90	
	LYS47	π-Alkyl	4.23	
	LEU79	π-Alkyl	5.47	
	ALA45	π-Alkyl	4.03	
	ALA163	π-Alkyl	5.04	
WEHI-345	ILE162	Hydrogen Bond	2.43	−7.50
	GLU66	Hydrogen Bond	3.06	
	THR95	π-Donor	4.03	
	ALA163	Alkyl	4.17	
	ALA163	Alkyl	3.09	
	LEU70	Alkyl	3.60	
	LEU79	Alkyl	3.87	
	LEU79	Alkyl	5.08	
	ILE69	Alkyl	4.98	
	LEU70	π-Alkyl	4.600	
	ALA163	π-Alkyl	5.19	
	VAL32	π-Alkyl	4.50	
	ALA45	π-Alkyl	4.48	
	LYS47	π-Alkyl	3.82	
	ILE69	π-Alkyl	4.63	

**Table 4:** Interactions between RIPK2 (from PDB ID 4C8B) and the following compounds are shown, after docking calculations: **ZINC90174766**, **ZINC91725665**, **ZINC69431616**, **ZINC12230819**, **ZINC12230826**, **ZINC12230756**, **ZINC69431621**, **ZINC90174764**, **ZINC91881108**, **ZINC69349687**, **ZINC91725663**, **ZINC89571615**, **ZINC87131463** and **ZINC81021663**.

Compound	Amino Acid	Type	Distance (Å)	Binding Affinity (in kcal/mol)
**ZINC90174766**	LEU70	π-Alkyl	5.12	−11.00
	ALA163	πAlkyl	4.77	
	LEU79	π-Alkyl	5.23	
	ALA45	π-Alkyl	3.71	
	VAL32	π-Alkyl	5.14	
**ZINC91725665**	VAL32	Alkyl	4.12	−10.80
	ALA45	Alkyl	3.04	
	LEU79	Alkyl	4.42	
	LYS47	π-Alkyl	4.55	
	ALA163	Alkyl	2.94	
	GLU66	Hydrogen Bond	2.54	
	ILE69	π-Alkyl	5.11	
	LEU70	π-Alkyl	4.37	
	LYS47	π-Alkyl	5.29	
**ZINC69349685**	LEU79	Alkyl	5.06	−10.40
	ALA163	π-Alkyl	5.48	
	ILE69	π-Alkyl	4.56	
**ZINC69431616**	VAL32	π-Alkyl	5.10	−10.20
	ALA45	π-Alkyl	3.65	
	LEU79	π-Alkyl	5.40	
	LEU70	π-Alkyl	4.28	
**ZINC12230819**	GLU66	Hydrogen Bond	2.71	−9.80
	ILE69	π-Alkyl	5.44	
	LYS47	Hydrogen Bond	2.79	
	LYS47	Hydrogen Bond	3.03	
**ZINC12230826**	LYS47	Hydrogen Bond	2.81	−9.80
	LYS47	Hydrogen Bond	2.64	
	GLU66	Hydrogen Bond	2.54	
	GLU66	Hydrogen Bond	3.32	
	HIS144	π-Alkyl	3.23	
**ZINC12230756**	LYS47	Hydrogen Bond	2.78	−8.50
	LYS47	Hydrogen Bond	2.33	
	GLU66	Hydrogen Bond	2.43	
	LEU70	Alkyl	4.82	
	ILE69	π-Alkyl	5.10	
	VAL32	π-Alkyl	5.04	
	ALA45	π-Alkyl	4.57	
	LYS47	π-Alkyl	4.55	
	LEU79	π-Alkyl	5.39	
**ZINC91881108**	ASP164	Hydrogen Bond	2.53	−8.10
	ILE162	Hydrogen Bond	2.18	
	GLU66	Hydrogen Bond	2.28	
	ALA45	Alkyl	3.84	
	ALA163	Alkyl	4.54	
	LEU70	Alkyl	5.24	
	LEU79	Alkyl	4.75	
	VAL32	Alkyl	4.31	
	LYS47	Alkyl	4.39	
**ZINC69349687**	ALA163	π-Alkyl	5.08	−8.00
	VAL32	π-Alkyl	4.78	
	ALA45	π-Alkyl	5.27	
	LYS47	π-Alkyl	4.84	
	LEU79	π-Alkyl	5.33	
	ALA163	π-Alkyl	5.45	
**ZINC89571615**	LYS47	Hydrogen Bond	2.52	−7.90
	GLU66	Hydrogen Bond	3.04	
	HIS144	Hydrogen Bond	2.70	
	GLU66	Hydrogen Bond	2.74	
	LEU70	Alkyl	4.93	
	ILE69	π-Alkyl	5.38	
**ZINC87131463**	HIS144	Hydrogen Bond	3.62	−7.90
	ILE69	Alkyl	4.17	
	LEU70	Alkyl	3.73	
**ZINC81021663**	ILE162	Hydrogen Bond	2.89	−7.80
	LYS47	Alkyl	5.02	
	LEU70	Alkyl	5.06	
	LEU79	Alkyl	5.20	
	ALA163	Alkyl	4.00	
	LEU70	Alkyl	4.26	
	LEU79	Alkyl	4.69	

**Table 5:** Similarity analyses for the molecular overlap of the compounds for 100ste, 100elt, 60est and 40elt, 50est/elt, relative to ponatinib.

Similarity of Overlay (%)					
Compound	100ste	100elt	60est/40elt	40est/60elt	50est/elt
**ZINC90174766**	0.7169	0.3917	0.4757	0.3669	0.4202
**ZINC91725665**	0.7128	0.4167	0.4802	0.3666	0.4234
**ZINC91881108**	0.4865	0.5520	0.4701	0.4775	0.4740

**Table 6:** Similarity analyses for the molecular overlap of the compounds for 100ste, 100elt, 60est and 40elt, 50est/elt, relative to WEHI-345.

Similarity of Overlay (%)					
Compound	100ste	100elt	60est/40elt	40est/60elt	50est/elt
**ZINC12230819**	0.7824	0.3623	0.4717	0.3390	0.4036
**ZINC91881108**	0.6491	0.5645	0.4611	0.4131	0.4361
**ZINC81021663**	0.6802	0.3527	0.5687	0.5192	0.5438

**Table 7:** Compounds with QFIT > 0, which were analyzed using the CHEMGPS-NP server.

Compound	QFIT Value
**ZINC91881108**	52.28
**ZINC81021663**	31.26
**ZINC89571615**	25.83
**ZINC69349687**	18.61
**ZINC12230819**	17.69
**ZINC69349685**	15.23
**ZINC12230756**	14.62
**ZINC69431616**	14.13
**ZINC12230826**	12.21

**Table 8:** Pharmacokinetic properties of nine selected compounds as well as ponatinib and WEHI-345.

Compound	Stars	Rule of Five	% HOA	ACP (nm/s)	AMP (nm/s)	QPlog Po/w	CNS	QPlog BBB
Ponatinib	1	1	73.645	67.076	119.394	4.605	1	−0.026
WEHI-345	0	0	93.941	456.487	211.950	3.313	−2	−1.220
**ZINC69349685**	0	0	100.00	1300.94	3123.35	2.563	−1	−0.281
**ZINC69431616**	0	0	100.00	1695.74	4585.45	2.910	−1	−0.145
**ZINC12230819**	0	0	80.392	362.468	467.713	1.305	−2	−1.048
**ZINC12230826**	0	0	82.927	462.942	598.190	1.413	−1	−0.871
**ZINC12230756**	0	0	84.316	561.255	418.589	1.395	−1	−0.809
**ZINC91881108**	0	0	94.985	1169.73	586.063	2.242	0	−0.577
**ZINC69349687**	0	0	100.00	1297.59	3147.44	2.566	−1	−0.282
**ZINC89571615**	0	0	83.225	229.628	131.490	2.395	−2	−1.455
**ZINC81021663**	0	0	89.971	946.013	748.108	1.667	0	−0.526

**Table 9:** Prediction of synthetic accessibility of ponatinib, WEHI-345 and the selected compound **ZINC91881108**.

Compound	Accessibility
Ponatinib	5.10
WEHI-345	4.86
**ZINC91881108**	5.01

**Table 10:** 3D Coordinates and pharmacophoric features of the best-ranked pharmacophoric model.

Pharmacophoric Feature	X	Y	Z	Radius (in Ǻ)
Hydrogen Bond Donor	3.71	−0.53	1.86	1.10
Hydrogen Bond Acceptor	2.47	−1.65	-0.38	1.11
Hydrogen Bond Acceptor	3.51	−0.10	1.79	1.26
Hydrophobic	−2.33	0.63	−0.57	0.55
Hydrophobic	0.68	1.00	1.43	1.42
